# *DAPK1* Promoter Methylation and Cervical Cancer Risk: A Systematic Review and a Meta-Analysis

**DOI:** 10.1371/journal.pone.0135078

**Published:** 2015-08-12

**Authors:** Antonella Agodi, Martina Barchitta, Annalisa Quattrocchi, Andrea Maugeri, Manlio Vinciguerra

**Affiliations:** 1 Department of Medical and Surgical Sciences and Advanced Technologies “GF Ingrassia”, University of Catania, Catania, Italy; 2 LaPoSS, Laboratory of Policies and Social Services, University of Catania, Catania, Italy; 3 University College London, Institute for Liver and Digestive Health, Royal Free Campus, London, United Kingdom; University of Torino, ITALY

## Abstract

**Objective:**

The Death-Associated Protein Kinase 1 (*DAPK1*) gene has been frequently investigated in cervical cancer (CC). The aim of the present study was to carry out a systematic review and a meta-analysis in order to evaluate *DAPK1* promoter methylation as an epigenetic marker for CC risk.

**Methods:**

A systematic literature search was carried out. The Cochrane software package Review Manager 5.2 was used. The fixed-effects or random-effects models, according to heterogeneity across studies, were used to calculate odds ratios (ORs) and 95% Confidence Intervals (CIs). Furthermore, subgroup analyses were conducted by histological type, assays used to evaluate *DAPK1* promoter methylation, and control sample source.

**Results:**

A total of 20 papers, published between 2001 and 2014, on 1929 samples, were included in the meta-analysis. *DAPK1* promoter methylation was associated with an increased CC risk based on the random effects model (OR: 21.20; 95%CI = 11.14–40.35). Omitting the most heterogeneous study, the between study heterogeneity decreased and the association increased (OR: 24.13; 95% CI = 15.83–36.78). The association was also confirmed in all the subgroups analyses.

**Conclusions:**

A significant strong association between *DAPK1* promoter methylation and CC was shown and confirmed independently by histological tumor type, method used to evaluate methylation and source of control samples. Methylation markers may have value in early detection of CC precursor lesions, provide added reassurances of safety for women who are candidates for less frequent screens, and predict outcomes of women infected with human papilloma virus.

## Introduction

Cervical cancer (CC) is the second most common cancer in women worldwide [1, 2]. The identification and treatment of women with cervical intraepithelial neoplasia (CIN) or carcinoma *in situ* (CIS), the precursor lesions of invasive CC, represent an important component of the prevention of CC [3]. CC arises by distinct morphologic changes from normal epithelium and progresses to carcinoma through a series of well-defined pre-invasive lesions. Histologically, CC presents as either squamous cell carcinoma (SCC) or adenocarcinoma (AC) [4], with SCC predominating. Persistence of human papilloma virus (HPV) is the main etiologic factor in the development of CC and the precursor lesions [5, 6]. However, only a small fraction of HPV-infected CIN lesions progress to invasive cancer, thus, other host factors play a role in cervical carcinogenesis [2, 7].

Among the putative molecular alterations involved in the neoplastic process, aberrant methylation might be a crucial event in the oncogenesis [8]. A recent meta-analysis confirmed that global DNA methylation levels, in tissues of several cancers, were significantly lower in cancer patients than in healthy controls [9]. Approximately 60% of all human promoters are associated with CpG islands. In the genome of untransformed cells, ~90% of all promoters are unmethylated [10]. Conversely, in cancer, the methylation of CpG regions of gene promoter is associated with inappropriate transcriptional repression and gene inactivation. Significantly, many of the inactivated genes are tumor suppressor genes [11,12] and the inhibition of these genes by methylation is implicated in cancer initiation, development, and progression [13]. Although it is difficult to establish whether such epigenetic alterations are causative or consequential of cancer, there is evidence that they can occur early in the neoplastic process [14]. Recently, the role of epigenetic mechanisms of gene inactivation has been examined in cervical oncogenesis [13,15–19].

Among the involved genes, the Death-Associated Protein Kinase 1 (*DAPK1*) gene has been frequently investigated in CC. DAPK1 is a novel 160 kd calmodulin-dependent serine/threonine kinase operating as a positive mediator of apoptosis, while apoptosis links to the development, progression, and metastasis of human cancer [20]. The DAPK1 C-terminal serine-rich tail peptide, which is conserved in death-domain-containing proteins, plays a negative regulatory role in the inhibition of DAPK1, whereas the removal of this region enhances the killing activity [21]. Hypermethylation of *DAPK1* has been frequently reported in various cancers types, including colon [22], head and neck [23], urinary bladder [24], lung [25–27], B cell lymphoma [28] and ovary [29]. In addition, it has been associated with the advanced stages of tumor development [30] and a poor prognosis in non-small cell lung carcinoma [31]. Since DAPK1 is a positive mediator of apoptosis, the silencing of *DAPK1* disabled the DAPK-mediated apoptosis and might then prompt metastasis in the cancer cells [32]. Furthermore, cells lacking *DAPK1* expression via promoter methylation became more invasive and metastatic [33].

In addition to the functional implications of gene inactivation in tumor development, genes that are frequently aberrantly methylated in specific tumours have been used as molecular targets for the detection of neoplastic cells in body fluids providing additional targets for non-invasive early diagnosis and for cancer monitoring [34–36]. Thus, developing a panel of methylation markers may have value in early detection of CC precursor lesions, provide added reassurances of safety for women who are candidates for less frequent screens, and predict outcomes of women infected with HPV [34].

The aim of the present study was to carry out a systematic review and a meta-analysis in order to summarize the current published studies and to evaluate *DAPK1* promoter methylation as an epigenetic marker for CC risk.

## Methods

### Search strategy and selection criteria

Firstly, a systematic literature search in the Medline database, using PubMed, was carried out for epidemiological studies, published before July 2014, investigating the association between gene promoter methylation and CC risk. Literature search was conducted independently by two Authors using the keywords “promoter methylation” and “cervical neoplasia”. The searches were limited to studies written in English; abstracts and unpublished studies were not included. Moreover, the reference lists from selected articles were checked to search for further relevant studies. The aim of the first selection was to identify studies that investigated the association between promoter methylation of any gene and CC risk; no studies were excluded a priori for weakness of design or data quality. Accordingly, articles were selected only if they satisfied the following criteria: i) case-control or cohort study designs, and ii) studies that assessed the association of gene promoter methylation and CC. Subsequently, since *DAPK1* gene has been identified as the most common analyzed and studied gene, a meta-analysis of articles reporting the association between *DAPK1* promoter methylation and CC risk was performed. Thus for inclusion in the quantitative analysis, studies had to meet the following criteria: i) studies that assessed the association between *DAPK1* methylation and CC and ii) provided data about the frequency of *DAPK1* methylation in cancer and in control groups. Furthermore, exclusion criteria were as follows: i) studies that did not use exfoliated cells, cervical biopsies or urines as samples and ii) in which control or cancer groups included individuals with various types of precancerous lesions. The preferred reporting items for systematic reviews and meta-analysis (PRISMA) guidelines were followed [37] (S1 and S2 Files).

### Data extraction and quality assessment

Two of the Authors independently reviewed all the eligible studies and abstracted the following information in a standard format: first Author’s last name, year of publication, country where the study was performed, sample type, experimental methods to assess *DAPK1* methylation and number of cases and controls subjects.

### Statistical Analysis

All data were analyzed using the Review Manager 5.2 software provided by the Cochrane Collaboration (http://ims.cochrane.org/revman).

Forest plots were generated to illustrate the study-specific effect sizes along with a 95% CI. The fixed-effects or random-effects models, according to heterogeneity across studies, were used to calculate the ORs and 95% CIs in order to assess the association between *DAPK1* promoter methylation and CC risk. Where a value of zero in the number of promoter methylation events caused problems with computation of the ORs for individual studies, the Review Manager 5.2 software provided to add a value of 0.5 to all cells of the related crosstab [38].

Heterogeneity across studies, was measured using the Q-test based on the χ2 statistic, considering significant statistical heterogeneity as p <0.1. As Cochran’s test only indicates the presence of heterogeneity and not its magnitude, we also reported the I^2^ statistic, which estimates the percentage of outcome variability that can be attributed to heterogeneity across studies. An I^2^ value of 0% denotes no observed heterogeneity, whereas, 25% is ‘‘low”, 50% is ‘‘moderate” and 75% is ‘‘high” heterogeneity [39]. We also estimated the between-study variance using tau-squared (t) statistic [40].

Furthermore, subgroup analyses were conducted by histological type (SCC and AC), by assays used to evaluate *DAPK1* promoter methylation (Methylation Specific PCR—MSP and real-time quantitative MSP—qMSP), and by control sample source (normal cervical tissues—NT and benign cervical tissues—BCT). A sensitivity analysis was performed to find relatively poor-quality studies by the omission of a single study at a time and to see whether a particular omission could affect the overall OR value and the heterogeneity across studies.

To determine the presence of publication bias, the symmetry of the funnel plots in which ORs were plotted against their corresponding standard errors were assessed.

## Results

### Search results and data characteristics

**Fig 1:**
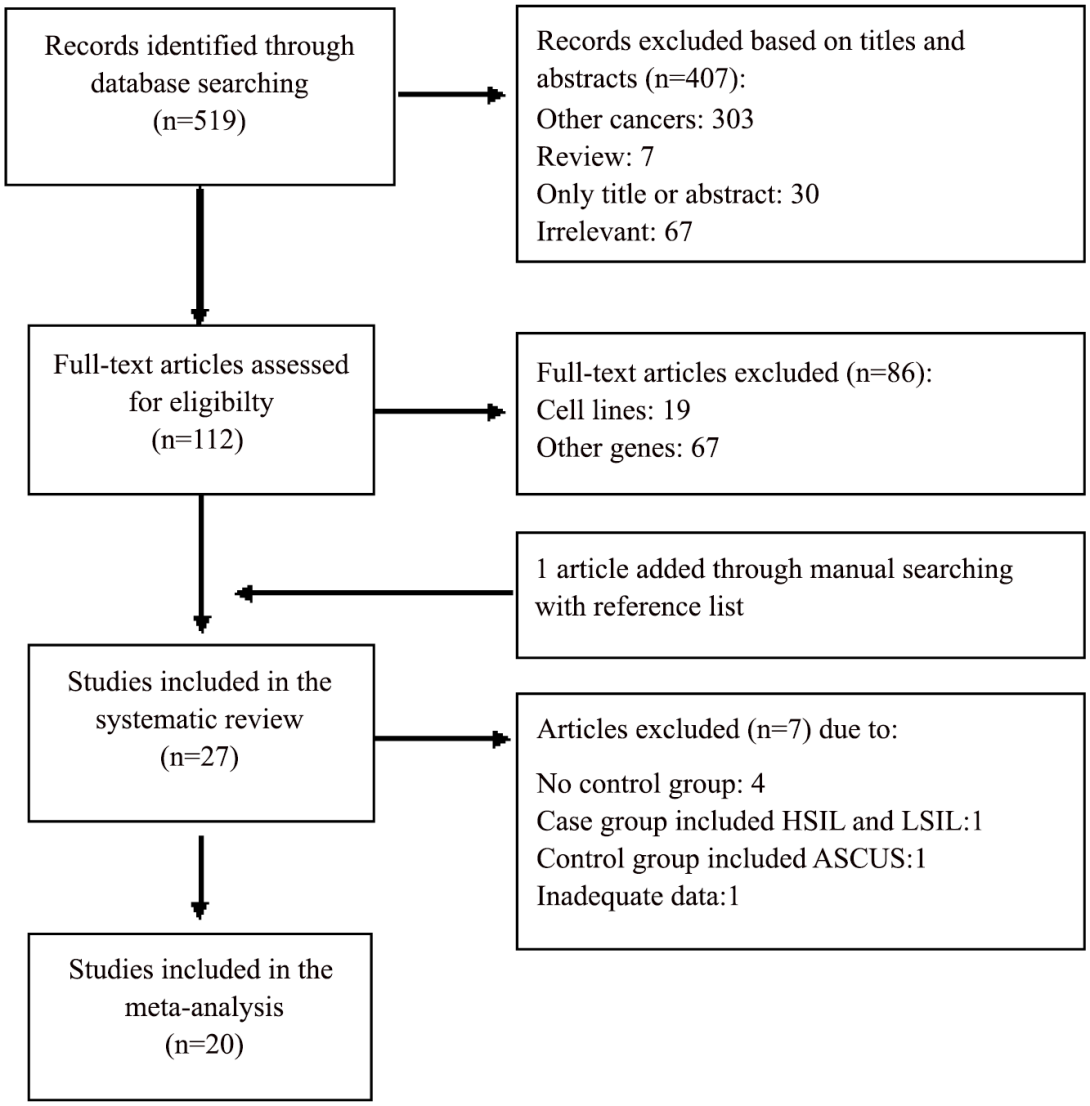
Flow diagram of study selection.

The detailed steps of the systematic review and meta-analysis process are given as a PRISMA flow chart (Fig 1). A total of 519 articles were retrieved from the database. After exclusion of studies that not met the inclusion criteria, *DAPK1* resulted the most common analyzed gene. Subsequently, one article was added through manual searching with reference list and thus 27 papers, published between 2001 and 2014, were included in the systematic review and summarized in Table 1. A total of 13 studies were from Asian countries (48%) [13, 17, 36, 41–50], 6 from European countries (22%) [3, 16, 51–54], 5 from USA (19%) [55–59] and 3 from Africa (11%) [60–62]. All studies evaluated *DAPK1* promoter methylation in SCC and 12 studies (44.4%) also in AC. Regarding the method of promoter methylation evaluation, the “gold standard method”, used in most studies (67%), was MSP, followed by qMSP (26%), sequencing (3.5%) and Methylation specific-multiplex ligation-dependent probe amplification (MS-MLPA) (3.5%).

**Table 1:** Studies included in the systematic review and in the meta-analysis.

Reference	Author	Year	Country	Method	Source of cancer sample	Source of control	Methylation Tumor	Methylation Control	Note
43	Banzai et al.	2014	Japan	MSP	Biopsy	NT	40/53	1/24	
41	Dong et al.	2001	Korea	MSP	Biopsy	BCT	27/53	0/24	
61	Feng et al.	2007	Senegal	qMSP	Urine	NT	31/63	1/16	
60	Feng et al.*	2005	Senegal	MSP	Biopsy		50/91	3/140	Control group included ASCUS
51	Flatley et al.	2009	UK	MSP	Scrape	NT	17/42	0/40	
59	Gustafson et al.*	2004	USA	MSP	Scrape		NA	NA	Case group included LSIL and HSIL
52	Henken et al.*	2007	Netherlands	MS-MLPA	Biopsy		NA	NA	No control group
50	Huang et al.	2011	Taiwan	MSP	Scrape	NT	13/26	3/15	
16	Iliopoulos et al.	2009	Greece	qMSP	Biopsy	NT	41/61	0/15	
13	Jeong et al.	2006	Korea	MSP	Biopsy	BCT	35/78	1/24	
58	Kahn et al.*	2008	USA	qMSP	Scrape		NA	NA	No control group
56	Kalantari et al.*	2014	USA	Sequencing	Biopsy		NA	NA	Inadequate data
46	Kang et al.	2005	Korea	MSP	Biopsy	BCT	60/82	0/17	
48	Kang et al.*	2006	Korea	MSP	Biopsy		NA	NA	No control group
49	Kim et al.	2010	Korea	MSP	Scrape	BCT	50/69	11/41	
44	Leung et al.	2008	China	MSP	Biopsy	AT	60/107	0/27	
62	Missaoui et al.	2011	Tunisia	MSP	Biopsy	BCT	10/14	0/8	
55	Narayan et al.	2003	USA	MSP	Biopsy	NT	37/82	0/8	
47	Niyazi et al.	2012	China	MSP	Biopsy	BCT	19/30	1/30	
3	Reesink-Peters et al.	2004	Netherlands	qMSP	Scrape	NT	35/48	2/41	
57	Shivapurkar et al.	2007	USA	qMSP	Biopsy	BCT	24/45	0/12	
36	Sun et al.	2012	China	MSP	Scrape	NT	11/14	157/336	
53	Wisman et al.	2006	Netherlands	qMSP	Scrape	BCT	13/28	0/19	
45	Yang et al.	2004	China	MSP	Biopsy	AT	51/85	0/100	
55	Yang et al.	2010	Netherlands	qMSP	Biopsy	BCT	31/60	5/20	
42	Yang et al.*	2006	China	MSP	Biopsy		NA	NA	No control group
17	Zhao et al.	2008	China	MSP	Biopsy	BCT	34/52	0/20	
	Total						639/1092	182/837	Studies included in meta-analysis

* studies excluded from meta-analysis

MSP: Methylation Specific PCR;

qMSP: quantitative real-time MSP;

NT: Normal cervical Tissue;

BCT: Benign Cervical Tissue;

AT: normal cervical tissues adjacent to the tumor;

ASCUS: Atypical Squamous Cells of Undetermined Significance;

LSIL: Low-grade Squamous Intraepithelial Lesion;

HSIL: High-grade Squamous Intraepithelial Lesion.

### Meta-analysis

Of the 27 selected articles, 4 studies conducted without a control group, 2 studies which included precancerous lesions in control or in case groups and 1 study which reported inadequate data, were excluded from the meta-analysis. Thus, 20 studies (74%) evaluating *DAPK1* promoter methylation both in tumor and in healthy control samples were included in the present meta-analysis. Overall, the studies reported results obtained from 1929 samples: 1092 from cancer patients and 837 from controls. Regarding the source of control samples, 10 studies evaluated *DAPK1* promoter methylation in BCT from patients having gynaecological diseases such as uterine myoma, adenomyoma, and uterine prolapse, 8 studies in NT from healthy people and 2 studies in normal cervical tissues adjacent to the tumor.

**Fig 2:**
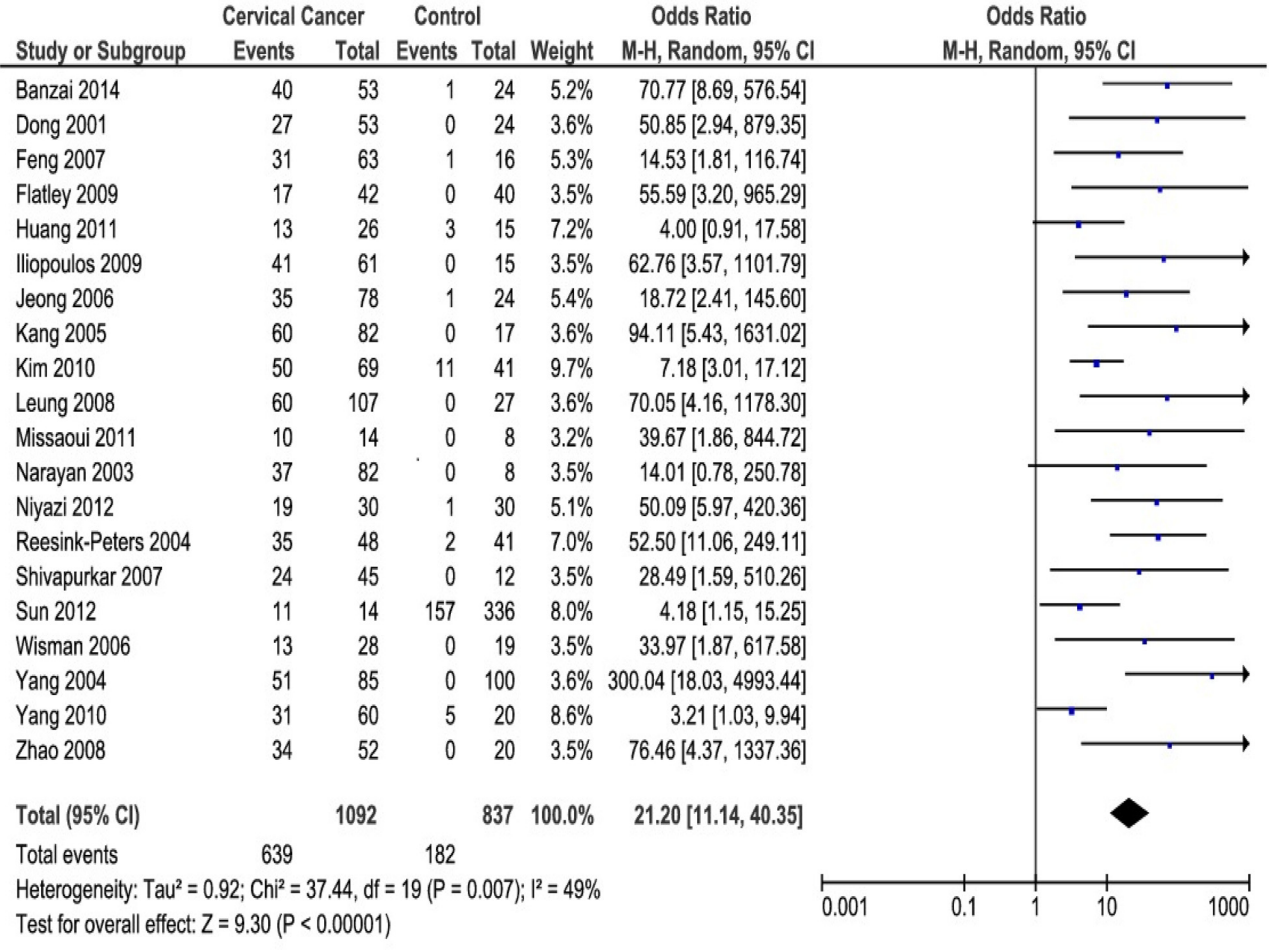
Forest plot of *DAPK1* promoter methylation and cervical cancer risk using random effects model.

*DAPK1* promoter methylation was associated with an increased CC risk with a pooled OR of 19.97 (95% CI = 13.57–29.38) based on the fixed effects model. However, due to the significant heterogeneity (I^2^ = 49%; p = 0.007), a pooled OR of 21.20 (95%CI = 11.14–40.35), based on the random effects model, was obtained (Fig 2). Subgroup analyses were performed by histological types, methods for methylation analysis and sources of control samples. The association between *DAPK1* promoter methylation and CC was confirmed in each subgroup (S1 Table).

In addition, the sensitivity analysis found the study by Yang et al. (2010) [54], as the relatively poor-quality study. When this study [54] was omitted, the between study heterogeneity decreased to I^2^ = 39% (p = 0.04), and the association between *DAPK1* promoter methylation and CC risk increased (OR: 24.13; 95% CI = 15.83–36.78) (S1 Fig)

**Fig 3:**
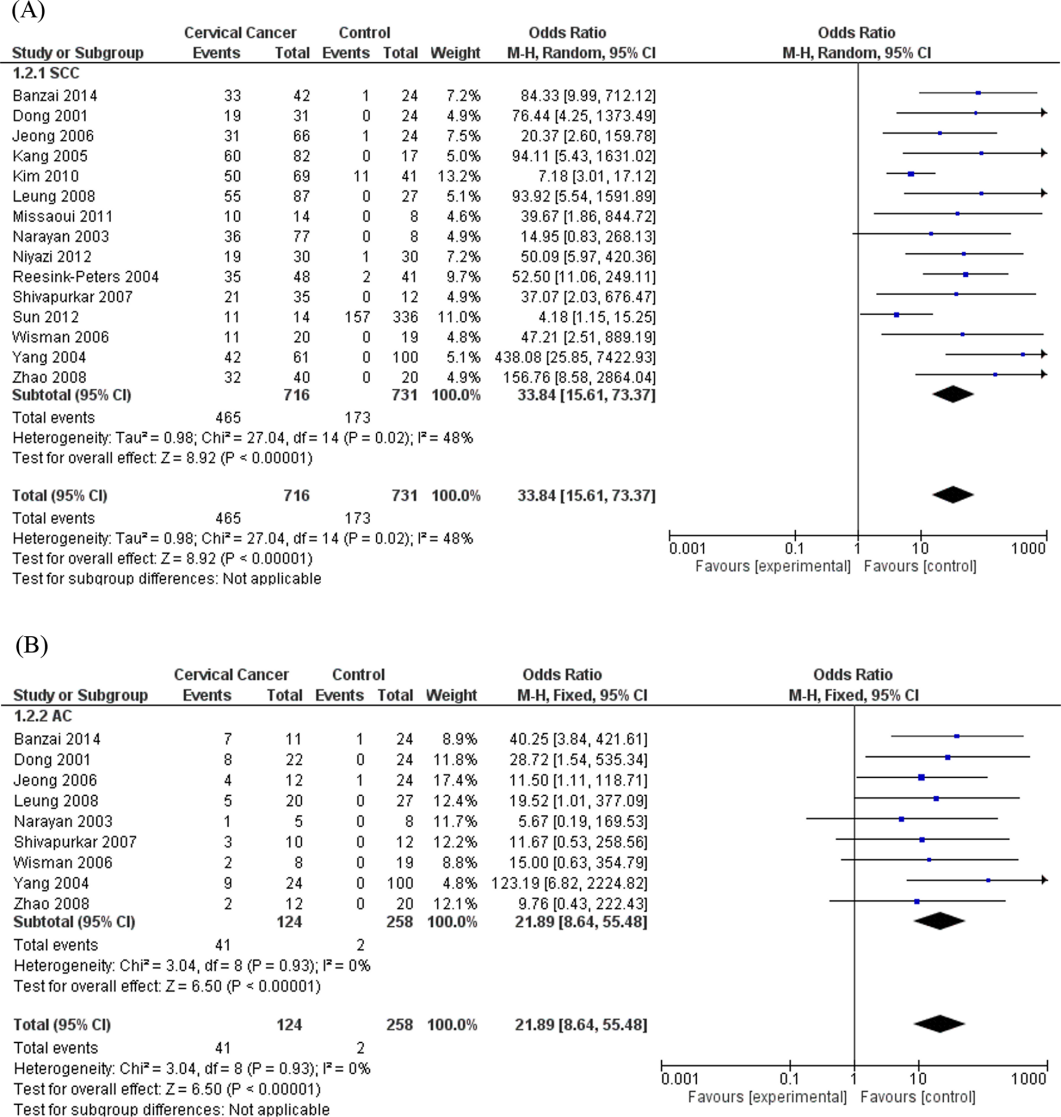
Subgroups analysis based on histological cancer type, omitting one heterogeneous study. (A) Squamous Cell Carcinoma (SCC) subgroup analysis, based on the random effects model. (B) Adenocarcinoma (AC) subgroup analysis, based on the fixed effects model.

**Fig 4:**
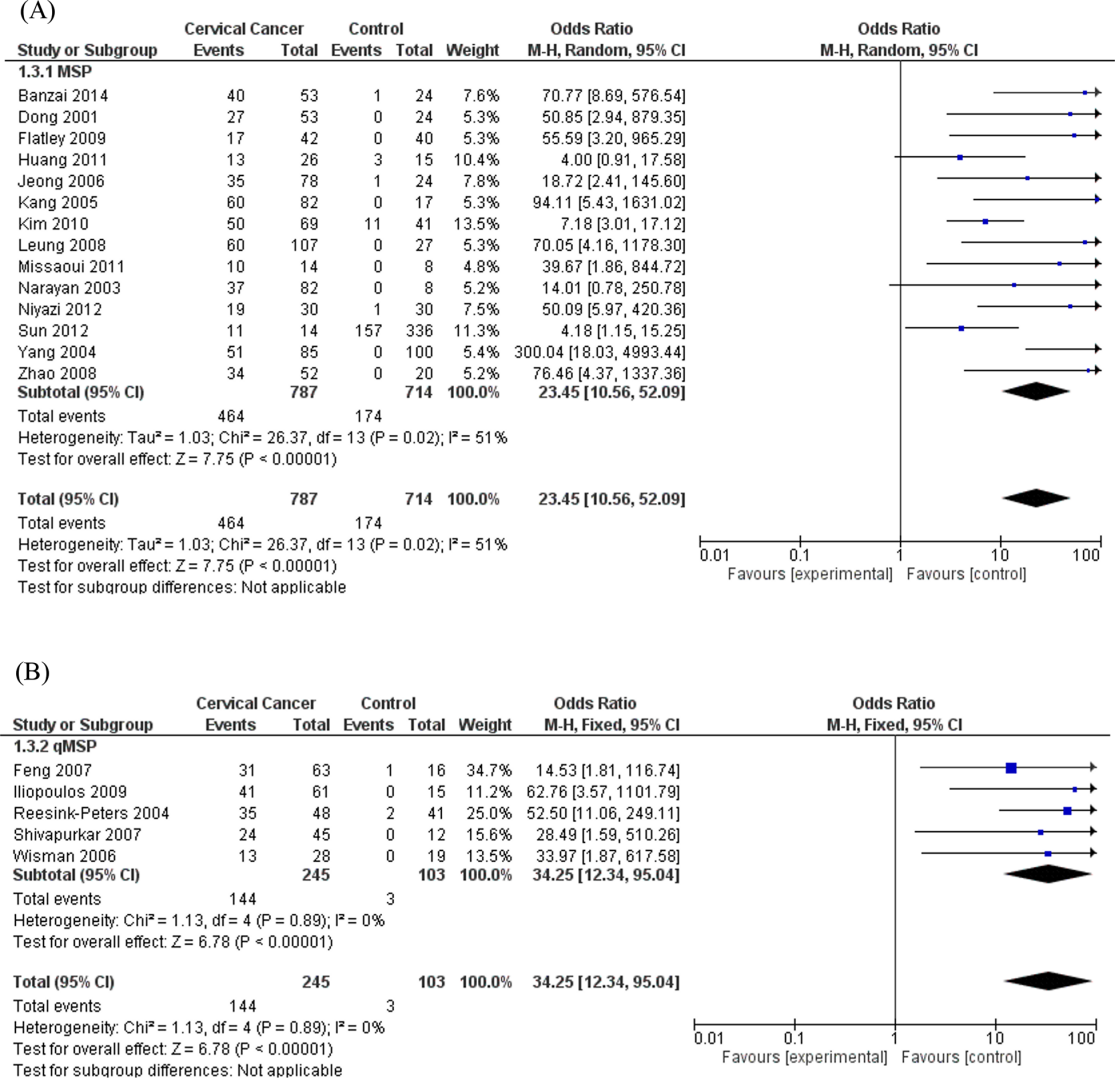
Subgroup analysis based on assays methods used, omitting one heterogeneous study. (A) Methylation-Specific PCR (MSP) subgroup analysis, based on the random effects model. (B) Quantitative real-time MSP (qMSP) subgroup analysis, based on the fixed effects model.

**Fig 5:**
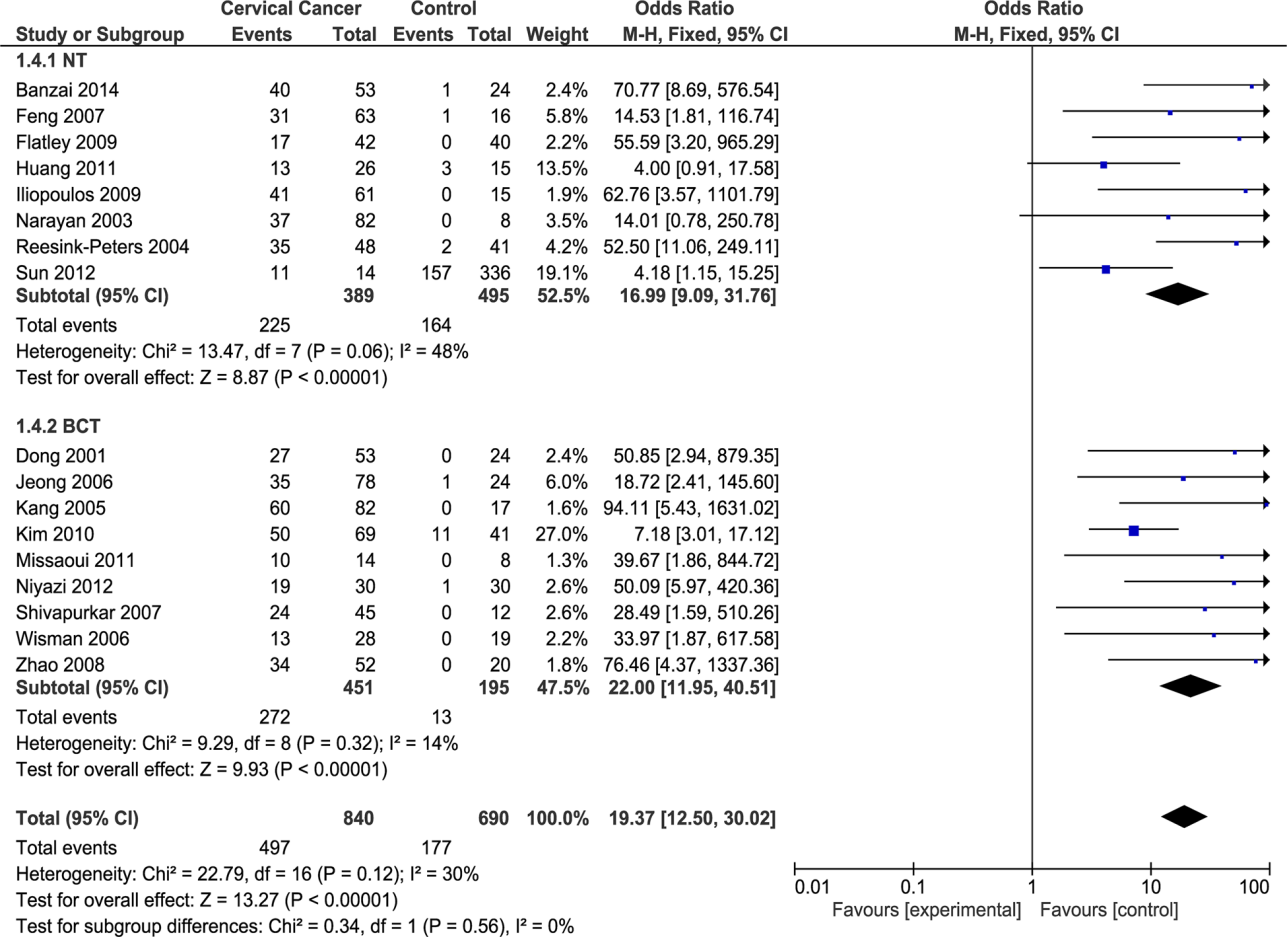
Subgroups analysis based on source of control sample, omitting one heterogeneous study [54]. NT: Normal cervical Tissue; BCT: Benign Cervical Tissue.

Subgroup analyses omitting the heterogeneous study [54] were performed. Subgroup analysis by histological types showed that the heterogeneity totally disappeared in AC subgroup (I^2^ = 0%; p = 0.93) and the association was confirmed both in SCC (OR = 33.84; 95% CI = 15.61–73.37; based on the random effects model) (Fig 3A) and AC (OR = 21.89; 95% CI = 8.64–55.48; based on the fixed effects model) (Fig 3B) subgroups. Furthermore, subgroup analysis based on assays methods used to evaluate *DAPK1* promoter methylation was performed including the two common techniques, MSP and qMSP. The ORs were 23.45 (95% CI = 10.56–52.09), based on the random effects model, in MSP subgroup, and 34.25 (95% CI = 12.34–95.04), based on the fixed effects model, in qMSP subgroup, while the I^2^ were 51% and 0%, respectively (Fig 4A and 4B). The subgroup analysis by source of control sample, and particularly between NT and BCT, reported that the ORs were 16.99 for NT (95% CI = 9.09–31.76) and 22.00 for BCT (95% CI = 11.95–40.51), respectively. Heterogeneity in NT and BCT subgroups were low with I^2^ = 48% and I^2^ = 14%, respectively (Fig 5).

**Fig 6:**
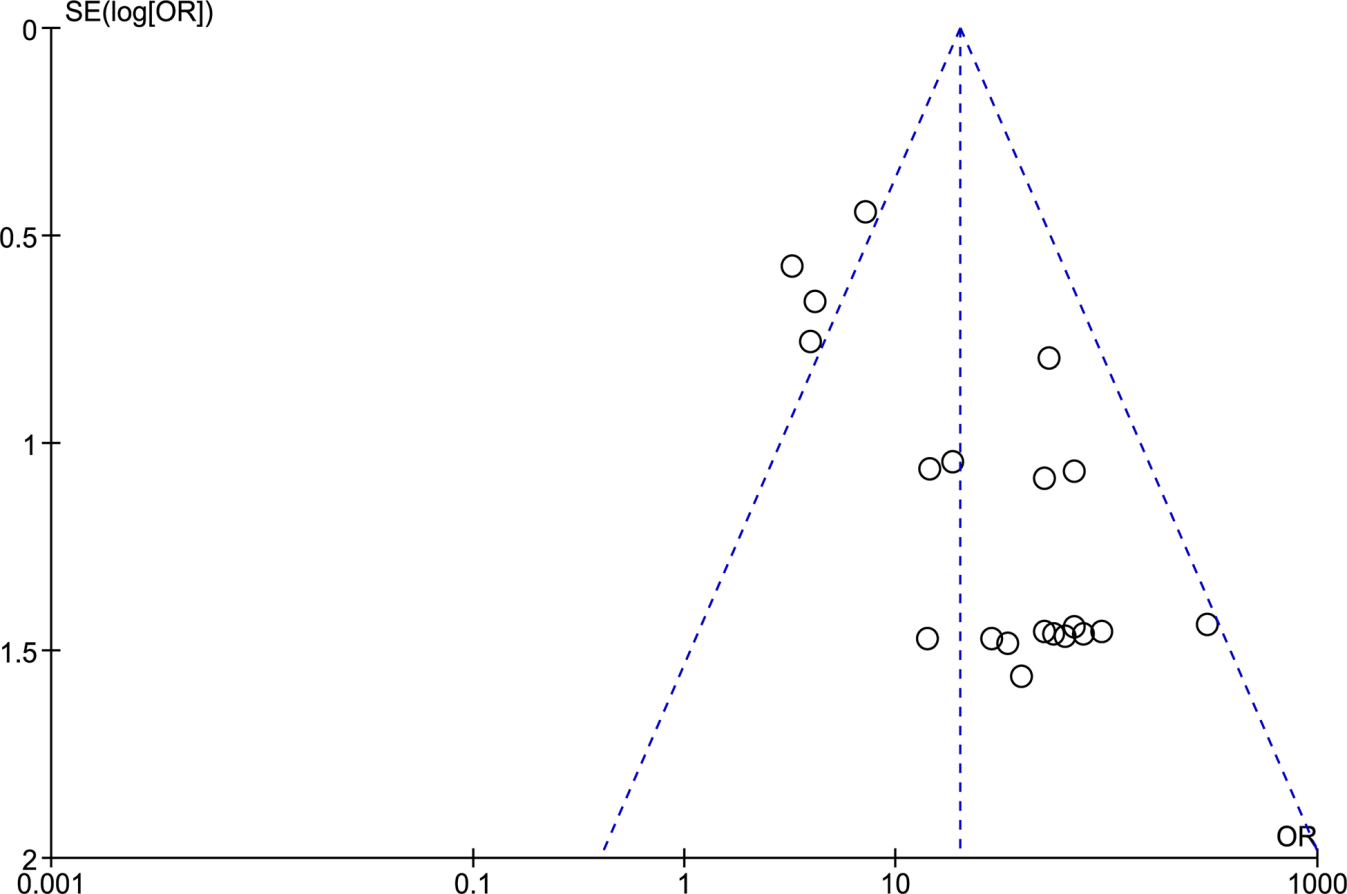
Funnel plot of *DAPK1* methylation and cervical cancer risk.

The funnel plot of the pooled analysis (Fig 6), which is quite symmetric, suggests no significant bias among the included studies, however the shapes of the subgroups analyses (S2, S3 and S4 Figs) indicate small to moderate asymmetry, therefore publication bias cannot be completely excluded as a factor of influence on the present meta-analysis.

## Discussion

Tumor suppressor genes belonging to different pathways, as cell adhesion, DNA repair, cell cycle checkpoint control and nuclear receptors, have been found to be hypermethylated in CIN and CC [41, 55, 60].

A previous review [63] summarized the results of 51 published studies on methylation analysis performed in cervical tissues and cells and proposed that the combination of *DAPK1*, *CADM1*, and *RARB* genes would appear the most promising methylated gene panel to obtain an appropriate performance for CC screening.

The recent meta-analysis by Xiong et al. [64], including 15 studies, suggested a strong association between *DAPK1* promoter methylation and CC (pooled OR = 19.66; 95%CI = 8.72–44.31) indicating that *DAPK1* promoter methylation may be a biomarker during cervical carcinogenesis.

Our study reports results of a more comprehensive meta-analysis and, taking into account that promoter methylation could be a tissue-specific event [65, 66], provides a subgroup analysis by histological tumor type. The present meta-analysis concerned 20 unique articles and, on a total of 1092 from cancer patients and 837 control samples, reports a significant pooled OR of 21.20. Because of the moderate heterogeneity between studies, a sensitivity analysis and subgroup analyses by histological tumor types, sources of control samples and assays used to evaluate *DAPK1* promoter methylation were performed. Interestingly, removing the most heterogeneous study [54], the association between *DAPK1* promoter methylation and CC risk increased (OR: 24.13) and was confirmed in SCC and AC subgroups with a heterogeneity between study of I^2^ = 48% and I^2^ = 0%, respectively.

The gold standard method of promoter methylation evaluation was MSP, in which PCR products are run on a gel, and the results are reported as methylated or unmethylated at the target DNA sequence. Consequently, this method does not allow the identification of partial levels of methylation, a feature which is extremely relevant both biologically and clinically. Thus, qMSP has been developed in recent years to overcome this limitation of conventional MSP. In fact, qMSP is reported to be more specific and more sensitive than conventional MSP and allows for high-throughput analysis, making it more suitable as a screening tool [67–69]. In the present meta-analysis, considering these two detection methods, both subgroups reported a significant association between *DAPK1* promoter methylation and CC. Although heterogeneity between studies stood moderately high in MSP subgroup (I^2^ = 51%), the heterogeneity in qMSP subgroup decreased to I^2^ = 0%.

Finally, the subgroup analysis by source of control sample revealed a significant association in both subgroups; the heterogeneity in NT and BCT subgroups was moderately low (I^2^ = 48% and I^2^ = 14%, respectively).

The present study has some limitations. The number of studies included in the meta-analysis is modest (n = 20). Moreover, since all studies included had a case-control design, it is not possible to clarify if *DAPK1* promoter methylation is an early cancer-causing aberration or an effect of carcinogenesis. Accordingly, the potential of DNA methylation measurements requires validation in retrospective studies, but ultimately in large prospective clinical studies [70].

In addition, although sensitivity analysis and subgroup analyses were performed, the pooled estimates should be interpreted with caution, due to the moderate heterogeneity across studies. Finally, the small to moderate asymmetry in the funnel plots, suggests that publication bias cannot be completely excluded.

The usefulness of *DAPK1* tumour suppressor gene hypermethylation as an epigenetic marker is under intense investigation in many different cancers, including CC and its precursor lesions and the present meta-analysis provides scientific evidences to this debate, showing a significant strong association between *DAPK1* promoter methylation and CC. This result was confirmed independently by histological tumor type, method used to evaluate methylation and source of control samples.

## Supporting Information

S1 Fig Sensitivity analysis of 20 studies with the fixed effects model. (TIF)

S2 Fig Funnel plot of subgroups analysis based on histological cancer type, omitting one heterogeneous study [54]. SCC: Squamous Cell Carcinoma; AC: Adenocarcinoma.
(TIF)

S3 Fig Funnel plot of subgroups analysis based on method, omitting one heterogeneous study [54]. MSP: Methylation-Specific PCR; qMSP: quantitative real-time MSP.
(TIF)

S4 Fig Funnel plot of subgroups analysis based on source of control sample, omitting one heterogeneous study [54]. NT: Normal cervical Tissue; BCT: Benign cervical Tissue.
(TIF)

S1 File PRISMA checklist. (PDF)

S2 File Meta-analysis on Genetic Association Studies Checklist. (PDF)

S1 Table Subgroups analyses based on histological cancer type, method and source of control sample. SCC: Squamous Cell Carcinoma; AC: Adenocarcinoma; MSP: Methylation-Specific PCR; qMSP: quantitative real-time MSP; M+: the number of subjects/samples with methylation; M-: the number of subjects/samples with no methylation; NT: Normal cervical Tissue; BCT: Benign cervical Tissue.
(TIF)
