# Supplementary figures and images for: DAPK1 Promoter Methylation and Cervical Cancer Risk: A Systematic Review and a Meta-Analysis
Source: PLoS One. 2015 Aug 12;10(8):e0135078. doi: 10.1371/journal.pone.0135078 (PMC4534406; doi:10.1371/journal.pone.0135078)

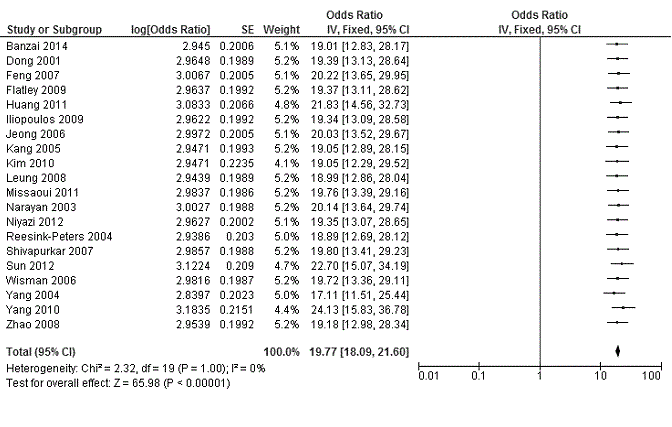

Supplement: S1 Fig — (TIF) [file pone.0135078.s001.tif]

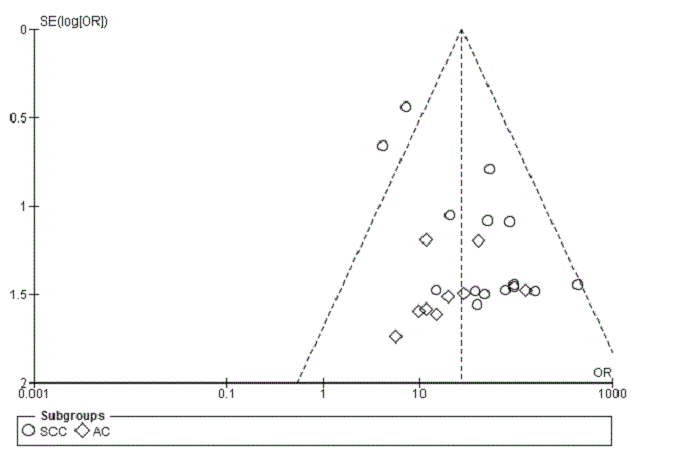

Supplement: S2 Fig — SCC: Squamous Cell Carcinoma; AC: Adenocarcinoma. (TIF) [file pone.0135078.s002.tif]

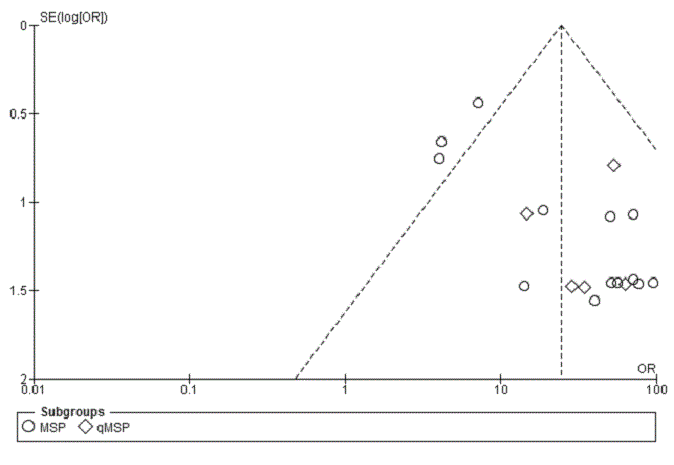

Supplement: S3 Fig — MSP: Methylation-Specific PCR; qMSP: quantitative real-time MSP. (TIF) [file pone.0135078.s003.tif]

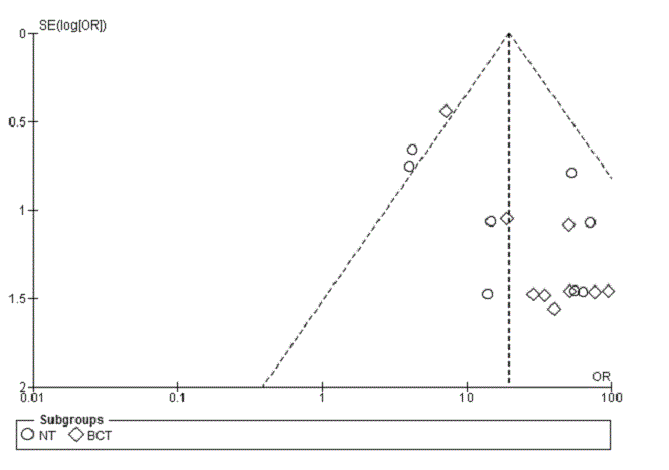

Supplement: S4 Fig — NT: Normal cervical Tissue; BCT: Benign cervical Tissue. (TIF) [file pone.0135078.s004.tif]

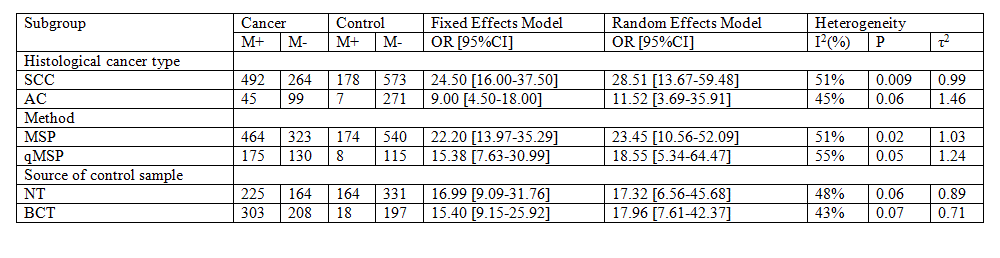

Supplement: S1 Table — SCC: Squamous Cell Carcinoma; AC: Adenocarcinoma; MSP: Methylation-Specific PCR; qMSP: quantitative real-time MSP; M+: the number of subjects/samples with methylation; M-: the number of subjects/samples with no methylation; NT: Normal cervical Tissue; BCT: Benign cervical Tissue. (TIF) [file pone.0135078.s007.tif]
