# Supplementary material for: DAPK1 Promoter Methylation and Cervical Cancer Risk: A Systematic Review and a Meta-Analysis
Source: PLoS One. 2015 Aug 12;10(8):e0135078. doi: 10.1371/journal.pone.0135078 (PMC4534406; doi:10.1371/journal.pone.0135078)
Supplement: S2 File — (PDF) [file pone.0135078.s006.pdf]

Meta-analysis on Genetic Association Studies Checklist | PLOS ONE

	Item	Section name and paragraph number within manuscript
	**Introduction**	
1	Provide a detailed justification for the polymorphism studied; if a single polymorphism was analyzed, give details as to why others were not included in the meta-analysis.	Introduction
2	Provide a detailed justification for the population(s) and clinical condition studied.	Introduction
	**Methods**	
3	Provide full details of the search strategy employed; outline the full electronic search strategy –specific combination of keywords and any limits applied- for at least one database. Specify whether synonyms of polymorphisms/genes (e.g. SNP number) were searched.	Methods: Search strategy and selection criteria
4	Report full details on the inclusion and exclusion criteria applied for selecting studies. *Please list the excluded articles and the reasons for exclusion of each article in a supplementary file.*	Methods: Search strategy and selection criteria
5	Provide details on how the quality of the studies included in the analyses was assessed.	Methods: Search strategy and selection criteria
6	Describe steps taken to contact study authors to identify additional studies and to request missing data.	Methods: Search strategy and selection criteria
7	Describe how environmental effects were adjusted for, if this adjustment was not conducted, outline the reasons for this.	Methods: Statistical Analysis
8	Describe the methods of handling heterogeneity/between-study variance.	Methods: Statistical Analysis
9	Describe how the Hardy-Weinberg equilibrium and linkage disequilibrium were assessed.	Not applicable
10	Describe and justify the choice of model for the analyses (per-allele vs per-genotype vs genetic model-free, random effects vs fixed effects).	Methods: Statistical Analysis

11	Describe whether a sensitivity analysis has been completed.	Methods: Statistical Analysis
12	Describe whether an assessment of the effects of population stratification has been conducted.	Methods: Statistical Analysis
13	Describe whether study-specific results have been assessed and if so the reasons for this (e.g. forest plot).	Methods: Statistical Analysis
	**Results**	
14	Include flow diagram for the studies included in the meta-analysis as the first figure for the manuscript	Results: Search results and data characteristics;
15	Report details on allele/genotype prevalence.	Not applicable
16	Report the effect size estimates and p values for each analysis.	Results: Meta-analysis
	**Discussion**	
17	Discuss the limitations of the meta-analysis, including genotyping errors/bias and publication bias.	Discussion
18	If the meta-analysis identifies an association within a subgroup of the population studied but not another, discuss the implications of these results, and if applicable the possibility of subgroup-specific publication bias.	Discussion
19	Discuss the suitability of the sample size employed to the research question and the power of the study.	Discussion
